# Effect of Zinc Supplementation on Body Composition of Duchenne Muscular Dystrophy Patients: A Quasi-Experimental Study

**DOI:** 10.1155/2024/5522139

**Published:** 2024-09-18

**Authors:** Thais A. Cunha, Karina M. Vermeulen-Serpa, Evellyn C. Grilo, Mário E. T. Dourado-Júnior, Breno G. P. Bezerra, Núbia R. S. M. Torres, Márcia M. G. D. Lopes, Lucia Leite-Lais, José Brandão-Neto, Sancha H. L. Vale

**Affiliations:** ^1^ Postgraduate Health Sciences Program Federal University of Rio Grande Do Norte, Natal, Rio Grande Do Norte, Brazil; ^2^ Department of Internal Medicine Federal University of Rio Grande Do Norte, Natal, Rio Grande Do Norte, Brazil; ^3^ Chemistry Institute Núcleo de Processamento Primário e Reúso de Água Produzida e Resíduos-Nupprar Federal University of Rio Grande Do Norte, Natal, Rio Grande Do Norte, Brazil; ^4^ Postgraduate Nutrition Program Federal University of Rio Grande Do Norte, Natal, Rio Grande Do Norte, Brazil; ^5^ Department of Nutrition Federal University of Rio Grande Do Norte, Natal, Rio Grande Do Norte, Brazil

## Abstract

**Background:**

The study hypothesized that zinc supplementation would increase or preserve lean body mass in Duchenne muscular dystrophy (DMD) patients. Therefore, we aimed to evaluate the body composition of DMD patients before and after zinc supplementation.

**Materials and Methods:**

The study is a clinical trial comprising 21 boys diagnosed with DMD. Dietary intake parameters were evaluated before zinc supplementation. Serum zinc levels, anthropometry, and body composition were measured in three moments, four months apart. The patients received 5, 10, or 15 mg of zinc bis-glycine supplementation according to age as an oral solution daily for four months. The sample was distributed into two groups according to serum zinc status: zinc deficiency (G1) or adequate zinc (G2).

**Results:**

There was a significant difference in lean body mass between the groups: zinc deficiency (G1) or adequate zinc (G2), at three times (*p*=0.041, 0.016, and 0.009, respectively). After oral zinc supplementation, serum zinc levels were not different between groups. We did not observe differences when associating the parameters between times and groups.

**Conclusion:**

Zinc supplementation was able to maintain lean body mass and fat mass in patients with DMD with previous deficiencies. Therefore, it is necessary to have a prior screening of serum zinc levels to observe changes after supplementation.

## 1. Introduction

Approximately 1 : 3500 males are affected by Duchenne muscular dystrophy (DMD), a severe X-linked recessive disorder that develops when the dystrophin protein is deficient or absent, leading to the weakness of the muscle membrane and subsequent degradation. In addition, adipose and fibrous tissue replaces muscle fibers, resulting in muscle fragility and weakness [1–5].

DMD is a severe neuromuscular dystrophy, with symptoms onset in early childhood and a typical loss of ambulation at 12 years of age [6]. Glucocorticoids are used as a standard treatment to prolong ambulation. However, they have adverse consequences, such as metabolic dysfunction and obesity [4].

Changes in body composition are side effects of the disease and drug treatment, with increased total fat mass (FM) and reduced lean body mass (LBM). Changes in nutritional status occur as the disease progresses, ranging from overweight/obesity to malnutrition. The main objective of nutrition therapy in this disease is to preserve LBM and avoid excessive weight gain in affected boys [4, 7, 8].

More than two billion people worldwide are deficient in one or more micronutrients, and zinc is one of these essential nutrients [9]. Skeletal muscles and bones contain a high zinc concentration, which is associated with LBM, fat-free mass (FFM), and body FM [10]. Zinc is essential for LBM synthesis, and when it is deficient, it has been shown to increase the energy cost of tissue deposition [10, 11].

Besides these important roles, zinc is essential for growth and development, reproduction and sexual maturation, tissue repair, immune function, and the functioning of cell membranes [12]. Additionally, zinc intake affects its bioavailability. A diet low in zinc increases mineral absorption in all age groups, where homeostatic mechanisms positively regulate zinc absorption and retention [11, 13]. Its supplementation can accelerate the synthesis of LBM [10].

Although there is limited information on the relationship between zinc and DMD, the positive effects of zinc supplementation in healthy children have been extensively documented in scientific literature and in our specific region [14]. Therefore, this study aimed to investigate the hypothesis that zinc supplementation would lead to an increase in LBM in DMD patients. Therefore, we aimed to evaluate the body composition of DMD patients before and after zinc supplementation.

## 2. Materials and Methods

### 2.1. Study and Ethical Aspects

This study is a clinical trial, composing a larger study entitled “Nutritional Intervention in Duchenne muscular dystrophy”, which has been approved by the Research Ethics Committee of the Federal University of Rio Grande do Norte (UFRN), Brazil (*n*: 1.754.017) and registered with the Brazilian Registry of Clinical Trials (ReBEC) under the code RBR-7cfdxm. Data were collected between February 2018 and March 2020 at the Neurology Outpatient Clinic of the Onofre Lopes University Hospital (HUOL), in Natal/RN. All patients and legal guardians provided written informed consent before enrolling in the study.

### 2.2. Sample Characterization and Selection Criteria

**Figure 1:**
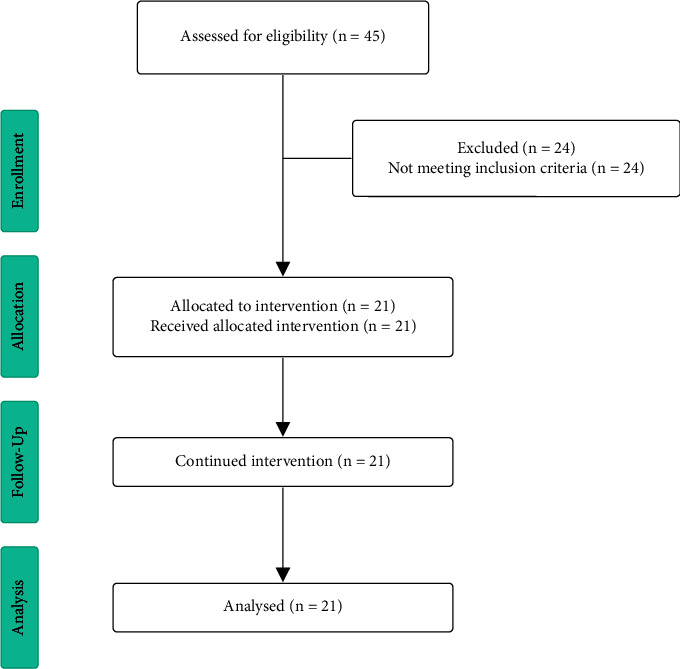
Flow diagram adapted from consolidated standards of reporting trials (CONSORT).

Given that DMD is a rare disease, the sampling process was not realized, and all patients diagnosed with DMD through genetic testing and seen in the Neurology Outpatient Clinic were included. A minimal age of five years was required as an inclusion criteria in this study, as it is the minimum age evaluated by dual-energy radiologic absorptiometry (DXA). Participants who were unable to provide blood samples on any of the three times were excluded. Participants who were unable to acquire measurements of weight and/or height due to physical restrictions or discomfort were also excluded from the study.  Figure 1 shows the flow diagram for clinical trials adapted by CONSORT.

### 2.3. Experimental Design

**Figure 2:**
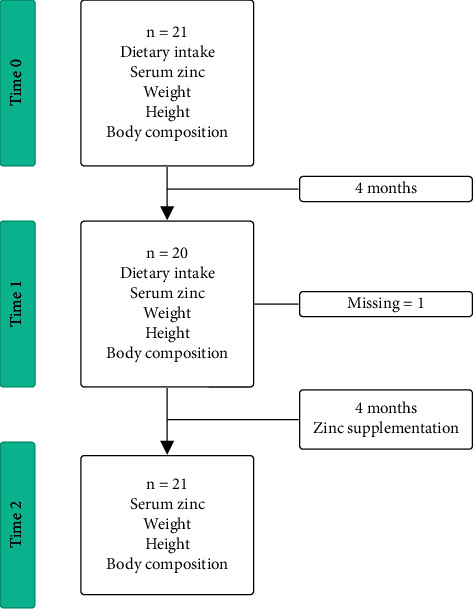
Study design.

This is a *quasi-experimental* study that includes prepost designs without a control group. Data on dietary intake, serum zinc, and anthropometric measurements such as weight and height were collected. In addition, patients underwent DXA to assess FM and LBM (Figure 2).

The evaluation started after the patients were allocated (T0) at the first consultation. After four months (T1), the patients returned to the outpatient clinic to be examined by a multidisciplinary team with the same criteria used in T0. Then, all patients used oral zinc supplementation during the following four months for a good action of this micronutrient on biological mechanisms. Dietary intake was not evaluated after supplementation (T2).

The sample was distributed into two groups according to serum zinc status: zinc deficiency (G1) or adequate zinc (G2). This distribution was performed to reduce the influence of greater zinc bioavailability after oral zinc supplementation in boys with previous low mineral intake [15].

At T1, one of the patients could not perform the DXA test because he presented respiratory distress in the dorsal decubitus position but underwent the other procedures.

### 2.4. Dietary Intake

Dietary intake was calculated from the analysis of two 24-hour food recalls (24-RH) at T0 and T1, performed according to Thompson and Byers [16]. Foods were converted into energy and nutrients using EasyDiet® (UFRN, Natal, Brazil). Afterward, data were exported to Microsoft Excel® software (Microsoft 365, 2019, 64 bit version, Microsoft Corporation, Washington, USA) and then added to Multiple Source Method® (MSM, version 1.0.1, German Institute of Human Nutrition, Potsdam-Rehbrücke, Germany), where the estimate of habitual nutrient intake was performed through correction for intrapersonal variability between each 24-HR [17].

The dietary intake of the boys was performed individually, where the adequacy of energy, fiber, and macronutrient intake was estimated, as well as the prevalence of inadequate zinc intake by the cut-off point method of estimated average requirements (EAR). The prevalence of inadequate zinc intake was determined from the percentage of patients whose usual intake is below the EAR [18–20]. The adequacy of energy intake was calculated based on the values in kcal/kg/day proposed by Davis, Samuels, and Mullins (2015), considering physical activity as sedentary [21]. Regarding macronutrients, raw intake values were compared with macronutrient distribution ranges (AMDR), and fiber was compared with adequate intake (AI) from dietary reference intakes (DRI) ranges for the healthy population, according to age group. The normal *Z* distribution determined the prevalence of inadequate zinc intake to obtain the percentage of patients whose usual intake was below the EAR [22].

### 2.5. Oral Zinc Administration

All participants received a shot solution of Taste-Free™ Zinc (zinc bisglycinate chelate, unflavored; Albion Laboratories, Utah, USA) orally, daily, and in the morning for four months. Zinc dosage varied according to age group, considering the data obtained from dietary intake and previous data related to zinc intake by children residing in the same city in this study [14]. In this way, the sum of zinc supplementation and zinc intake did not exceed the upper level described by the Institute of Medicine (IOM) (2001). Thus, children aged 5 to 8 years received 5 mg/day, children, and adolescents between 9 and 13 years received 10 mg/day, and adolescents over 14 years of age received 15 mg/day of supplementation.

In T1, zinc supplements were distributed, and supplementation compliance was monitored weekly via phone calls. In addition, the weekly count of remaining packages was documented, and the level of adherence to taking the supplements was 100%.

### 2.6. Serum Zinc

Blood samples were collected unfasted in the morning and stored at −80°C until analysis. The specimen's collection and processing were made following the International Zinc Nutrition Consultative Group's (IZiNCG) Technical Brief [23].

For analysis, the samples were thawed at room temperature and submitted to pretreatment for better zinc level determination. Serum zinc concentrations were determined by the inductively coupled plasma atomic emission spectrometer (ICP-OES) (model iCAP 6300 Duo, Thermo Fisher Scientific, Bremen, Germany), with the axial and radial views and a simultaneous charge injection device (CID) detector. This instrument is sensitive and commonly used for serum zinc analysis [24]. Argon with a purity of 99.996% (White Martins-Praxair, Natal, RN, Brazil) was used to purge optic plasma generation and was also used as a nebulization gas and auxiliary. The reference values of zinc concentration for males with blood collection in the morning and without fasting are 65 *μ*g dL (<10 years of age) and 70 *μ*g dL (≥10 years of age), according to IZiNCG (2012) [23]. Thus, these cut-off points were used to perform two groups based on serum zinc status immediately before supplementation, in T1: zinc deficiency (G1) or adequate zinc (G2).

### 2.7. Anthropometric and Body Composition Evaluation

Bodyweight (kg) and height (cm) were measured using an electronic ramp scale (KN R 500/50, KN Waagen, São Paulo, Brazil) and a stadiometer (Professional Sanny, American Medical do Brazil, São Paulo, Brazil), respectively. For those who could not stand, the recumbent height was measured. For screening overweight or underweight, the body mass index (BMI, in kg/m^2^) was calculated from the division of weight (kg) by the square of height (m^2^). The index BMI/Age (*z*-score) was calculated for those under 18 years old [25].

For the body composition evaluation (FM and LBM), DXA was performed with the GE Lunar DPX-NT Bone Densitometer (General Electric Company, WI, USA). Pediatric software (Lunar version 4.7, General Electric Company, WI, USA) or adult software (Lunar DPX-NT version 8, General Electric Company, WI, USA) was used according to participant age and handled by the same trained technician. The parameters of FM and LBM were evaluated and expressed as a percentage of body weight. Nutritionists performed anthropometric assessments.

### 2.8. Statistical Analysis

The data are presented as medians and interquartile ranges. Using the Shapiro–Wilk test, data normality was determined to be nonparametric. In order to analyze dietary intake, the nutrients were adjusted based on energy and presented as the mean and standard deviation, following the approach suggested by Willet and Stampfer [26]. The *t*-test of independent samples was used for zinc intake, and Cohen's *d* test was used to measure the effect size. The Friedman test was performed to analyze associations between age, BMI, and BMI/A between times, according to group. The Mann–Whitney test was performed to analyze associations between LBM (%) and FM (%) between groups if *p* < 0.05; Cohen's *r* effect size was applied [27, 28]. The associations between T0, T1, and T2 were analyzed with a split-plot ANOVA design, Wilks' Lambda test, and partial eta squared to determine the magnitude of the effect. Cohen's *r* and *d* consider effect sizes of 0.8, 0.5, and 0.2 to be large, medium, and small, respectively. All tests were performed using SPSS® software, version 28.0 (IBM SPSS Statistics for Windows, Version 28.0, IBM Corp., Armonk, NY, USA). A *p* value <0.05 was considered significant.

## 3. Results

Twenty-one patients, aged between 5.3 and 24.2 years, were evaluated. Among them, 71.4% did not have independent ambulation, and 81.0% used glucocorticoids (data not shown).  Table 1 shows the characteristics of patients according to time (T0, T1, and T2) in the total group and groups according to zinc status (G1 and G2).

**Figure 3:**
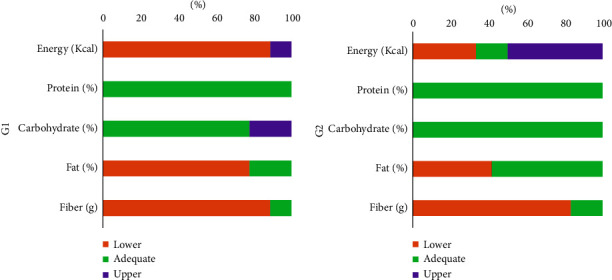
Prevalence of energy, macronutrients, and fiber intake inadequacy in Duchenne muscular dystrophy patients, according to zinc status group. (a) patients with zinc deficiency, (b) patients with adequate zinc.

Figure 3 shows the dietary intake of energy, macronutrients, and fibers in the groups. We observed in the zinc deficiency group (G1) a low intake of energy (88.8%), lipids (77.8%), fibers (88.9%), and adequate consumption of carbohydrates (77.8%) and proteins (100%).

In the adequate zinc group (G2), low fiber consumption (83.3%), adequate consumption of carbohydrates (100%), proteins (100%), lipids (58.3%), and high energy consumption (50%) were observed.

On average, patients consume more zinc than recommended by IOM (2001) [18]. However, it is possible to identify the inadequacy more prevalent in young patients (Table 2). Even though there was no statistical difference in zinc intake between the groups, these results showed a large effect size.

**Figure 4:**
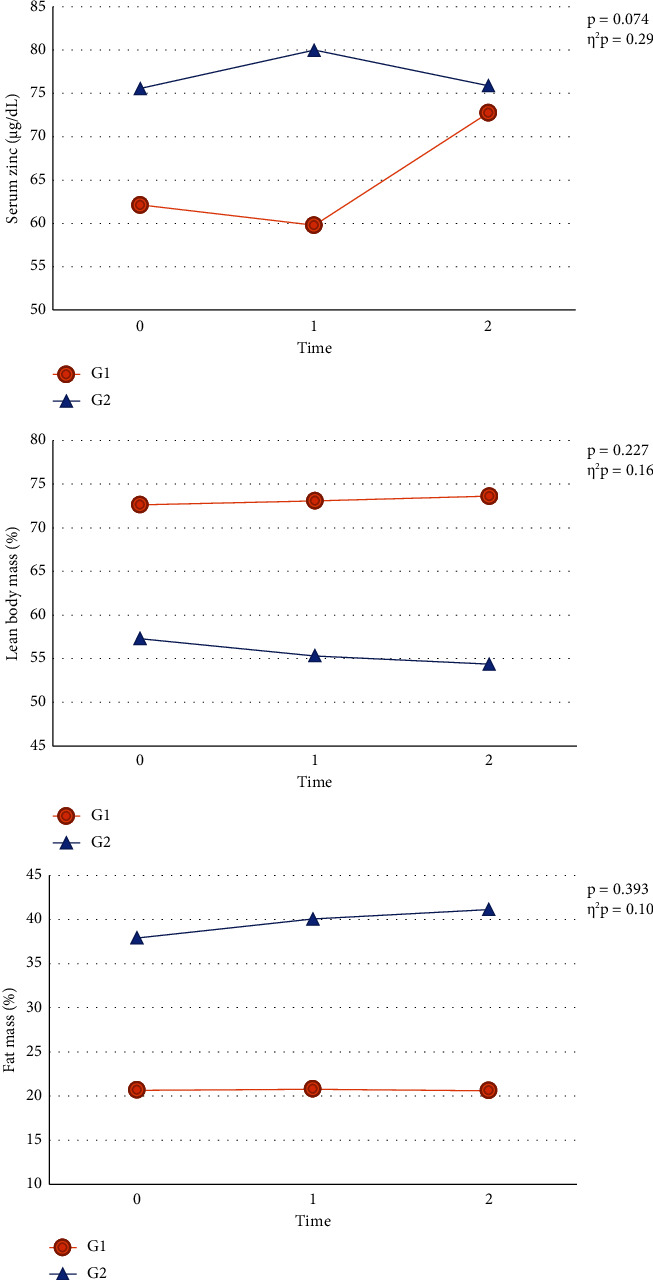
Association between the variables and time, according to the group. G1: patients with zinc deficiency; G2: patients with adequate zinc.

Serum zinc, FM, and LBM data are described according to time and group in Table 3. The total group was shown, and the difference in proportion between G1 and G2 was performed. After supplementation, we found that there was no longer a statistically significant difference in serum zinc levels between the groups. The primary aim was to examine the effects of zinc supplementation on the entire group, both before and after the intervention, without any subgroup divisions. However, when we categorized the participants into smaller groups based on their prior serum zinc deficiency status, we noticed notable differences in blood parameters (specifically serum zinc levels) and body composition (including lean body mass and fat mass). In addition, this classification took into account various factors that could impact the outcomes, including the evaluation of regular food consumption, blood levels, and the extent of zinc absorption after oral supplementation in the participants of the study. The association between times and groups is shown in Figure 4. In Table S1, we described FM and LBM in kilograms (kg), in the three times.

## 4. Discussion and Conclusion

This study broadly evaluated the body composition of DMD patients who were supplemented with zinc, also showing that 43% of patients had zinc deficiency before zinc supplementation (preintervention), and the younger patients consume fewer foods that contain zinc.

There is no difference in the inadequacy of zinc intake observed when comparing age groups. Still, the large effect size suggests that it is essential to pay attention to the consumption of zinc sources in children between 4 and 13 years of age in clinical practice [29, 30].

The total group had an average energy intake of 1,826 Kcal/d, which is considered a high value for sedentary people. However, after group analysis, G1 patients consume less energy than is recommended for their age group, and the opposite occurs in G2. Elliott et al. found an average of 1,645 kcal/d in boys with DMD with a mean age of 8.44 (1.90) [31]. Okada et al. found energy intake between 1,100 and 1,300 kcal/d in patients with progressive muscular dystrophy [32].

The initial cause of excess weight in DMD is unclear due to little knowledge about energy metabolism in this disease [31]. Glucocorticoids have significant side effects and start between 4 and 8 years of age [21]. For boys who use it, the percentage of FM can reach 70% [7]. However, it should be noticed that weight gain can be independent of the use of this drug [21]. In our study, 81.0% of DMD patients used glucocorticoids, and the literature shows its effects, such as weight gain, changes in bone health, behavioral problems, delayed puberty, short stature, and reflux [21, 33]. As most boys use it, we cannot rule out its influence on weight.

In the G2, 50.0% of the patients showed an energy intake higher than recommended. In this sense, the literature predicts that reducing mobility and physical activity decreases total energy expenditure. And this causes a food intake probably above the energy requirements [8].

In a study that evaluated the body composition of DMD patients using DXA, the linear regression model predicted a 5% annual increase in FM [34]. Previously, our research team evaluated DMD patients in a cross-sectional study based on anthropometric and bioelectrical impedance measurements and found that FM increased with age and was more prevalent in older patient groups [35]. However, in our study, using DXA, we assessed differences in body composition parameters between patients with zinc deficiency and those with adequate serum zinc levels.

Skeletal muscle metabolism is a major determinant of resting energy expenditure, and it is altered in neuromuscular diseases; boys with DMD lose 75% of their muscle mass by age 10. However, there is no evidence that boys with DMD should consume additional protein; protein intake should be recommended according to age [7, 21, 36]. All participants in this study had adequate protein intake for their age. In the same way, Okada et al. (1992) compared boys with DMD to healthy controls and found that DMD patients consumed less protein (39 to 47 g/day) than healthy controls [32].

Marginal serum zinc deficiency, either alone or in conjunction with low protein consumption, is linked to reduced lean body mass (LBM), diminished appetite, and excessive adiposity. However, these effects can potentially be modified or reversed through zinc supplementation [37, 38]. Zinc supplementation may not have been enough to change body parameters between groups in our study. Nonetheless, these body composition parameters were maintained (Figure 4).

We also observed that serum zinc in the group without prior zinc deficiency (G2) did not increase after supplementation (Figure 4). This may occur because zinc does not have a storage compartment in the human body and must be continuously replenished by dietary intake [10, 13].

Furthermore, the fractional absorption of dietary zinc in humans ranges from 16 to 50% and is inversely proportional to its oral intake. Zinc absorption is regulated by body homeostasis, which varies according to individual zinc status, and is more efficient than low-zinc diets [13]. This may explain the more efficient absorption of zinc in patients with previously lower serum levels.

Studies indicate that low blood zinc levels and decreased zinc intake are associated with an increase in obesity prevalence. However, the relationship between zinc and adipose tissue in obesity is still limited [38–40].

G2 showed smaller amounts of LBM and greater amounts of FM when compared to G1 (Figure 4), and this stimulatory effect may be related to the role of zinc in lipogenesis [41]. A study indicated that long-term zinc supplementation induced the accumulation of visceral adipose tissue in mice, independent of lipogenesis and lipolysis; and in another study, it increased the percentage of body fat in both genetic and diet-induced obese mice [10, 42, 43]. Also, zinc status in the body is related to appetite [41], which we can observe in G1, which has low energy and lipid intake.

Zinc supplementation affects body composition with increased LBM, this increase occurs mainly in children with preexisting growth deficiencies. Gunanti et al. concluded that the effect of zinc supplementation on body composition might not be consistent due to heterogeneity between studies [38].

Although we did not observe significant results after supplementation, we observed body composition maintenance in G1 (Figure 4), a stimulating outcome for this population as the disease progresses.

This study had limitations, such as not finding statistical formulas to analyze small groups studying rare diseases; the intervention period of four months may not have been long enough to see significant values of changes in body composition between times; and because it is a rare disease, we did not compare it to a control group.

In conclusion, the measurement and assessment of body composition in patients with DMD showed a distinct pattern. Therefore, zinc supplementation was unable to increase LBM as hypothesized, but it did maintain LBM and FM in these patients with previous serum zinc deficiencies. Consequently, a prior screening of serum zinc levels could be required to observe changes after supplementation.

## Figures and Tables

**Table 1:** Characteristics of Duchenne muscular dystrophy patients according to time.

Groups	Variables	T0	T1	T2	*p* value^∗^
Total group *n* = 21	Age, years	12.10 (9.00–18.05)	12.40 (9.35–18.70)	12.80 (9.75–19.05)	**<0.001**
	BMI/A, z-score	0.56 (−1.49–2.41)	0.57 (−1.86–2.53)	0.56 (−1.74–2.68)	0.551
	BMI, kg/m^2^	23.78 (18.96–25.60)	24.69 (18.71–25.78)	24.34 (17.96–25.45)	0.247

G1 *n* = 9	Age, years	13.40 (11.80–18.05)	13.80 (12.10–18.70)	14.10 (12.50–19.05)	**<0.001**
	BMI/A, z-score	−1.85 (−5,50−0,22)	−2.41 (−5.08–0.21)	−2.12 (−4.86–−0.07)	0.772
	BMI, kg/m^2^	19.00 (15.70–22.20)	18.70 (15.60–21.80)	18.00 (14.40–21.50)	0.135

G2 *n* = 12	Age, years	10.30 (7.70–18.43)	10.30 (8.08–18.73)	10.70 (8.43–19.03)	**<0.001**
	BMI/A, z-score	1.68 (0.66–3.10)	2.18 (0.98–3.34)	2.51 (0.74–3.34)	0.690
	BMI, kg/m^2^	24.80 (23.80–26.40)	24.80 (24.70–26.70)	25.40 (24.30–25.50)	0.717

G1: patients with zinc deficiency: G2: patients with adequate zinc. BMI/A: body mass index-for-age; BMI: body mass index; T0: time 0; T1: time 1; T2: time 2. Results are shown as medians and interquartile ranges (25–75%). ^∗^Friedman test. Bold values are statistically significant (*p* < 0.05).

**Table 2:** Daily nutritional recommendations, intake, and prevalence of inadequate zinc intake in patients with Duchenne muscular dystrophy.

Zinc	Age (years)^&^				*p* ^∗^	Effect size^┼^
	4–8 (*n* = 5)	9–13 (*n* = 9)	14–18 (*n* = 2)	19–30 (*n* = 5)		
EAR, mg	4.0	7.0	8.5	9.4	—	—
Intake, mg^∗∗^	7.3 (1.2)	11.5 (1.5)	15.6 (1.8)	21.4 (1.9)	—	—
Inadequacy, %	34.0	11.0	1.0	0.0	0.077	1.09

EAR: *estimated average* requirement; ^&^The age ranges are under the intervals recommended by IOM (2001). ^∗^*t*-test of independent samples. ^∗∗^Mean and SD. ^**┼**^: Cohen's *d* test.

**Table 3:** Serum zinc and body composition characterization in Duchenne muscular dystrophy patients, comparison between serum zinc status groups.

Time	Variables	Total group *n* = 21	G1 *n* = 9	G2 *n* = 12	*p* value^∗^	Effect size^∗∗^
T0 baseline	Serum zinc, *µ*g/dL	74.00 (56.00–82.00)	62.00 (49.00–76.00)	77.00 (74.00–84.00)	0.062	0.51
	LBM, %	71.88 (44.51–77.00)	75.52 (64.84–82.23)	51.36 (41.28–72.95)	**0.041**	0.31
	FM, %	21.21 (14.54–49.31)	15.32 (9.99–30.85)	44.88 (17.79–54.10)	**0.023**	0.35

T1 preintervention	Serum zinc, *µ*g/dL	68.00 (61.00–76.00)	61.00 (58.00–63.00)	76.00 (70.00–85.00)	**<0.001**	0.97
	LBM, %	69.24 (46.56–78.19)	77.47 (64.24–83.09)	50.89 (40.13–73.07)	**0.016**	0.20
	FM, %	24.60 (15.03–49.59)	15.73 (10.03–30,05)	44.13 (20.86–56.37)	0.069	0.12

T2 postintervention	Serum zinc, *µ*g/dL	74.00 (68.00–80.00)	74.00 (61.00–80.00)	75.00 (69.00–82.00)	0.644	0.13
	LBM, %	66.70 (45.85–79.35)	78.46 (61.66–85.20)	47.08 (41.46–68.42)	**0.009**	0.32
	FM, %	31.39 (15.16–49.90)	15.59 (7.89–31.12)	46.52 (29.19–53.74)	**0.010**	0.43

LBM: lean body mass; FM: fat mass; G1: patients with zinc deficiency; G2: patients with adequate zinc. Results are shown as median and interquartile ranges (25–75%). ^∗^Mann–Whitney test (G1 vs G2). ^∗∗^Cohen's *d* test: small >0.2; medium >0.5; and large >0.8. Bold values are statistically significant (*p* < 0.05).

## Data Availability

The data that supports the findings of this study are available in the supplementary material of this article.

## Supplementary Materials

Supplementary Materials The supplementary file (Table S1) provides a detailed description of the body composition analysis in kilograms (kg) of patients with Duchenne muscular dystrophy. The analysis compares the results based on the patients' previous serum zinc status, which is categorized into three groups.
