# Supplementary material for: Effect of Zinc Supplementation on Body Composition of Duchenne Muscular Dystrophy Patients: A Quasi-Experimental Study
Source: J Nutr Metab. 2024 Sep 18;2024:5522139. doi: 10.1155/2024/5522139 (PMC11424870; doi:10.1155/2024/5522139)
Supplement: Supplementary Materials — The supplementary file (Table S1) provides a detailed description of the body composition analysis in kilograms (kg) of patients with Duchenne muscular dystrophy. The analysis compares the results based on the patients' previous serum zinc status, which is categorized into three groups. [file 5522139.f1.docx]

**Table S1.** Body composition characterization in Duchenne muscular dystrophy patients, comparison between serum zinc status groups.

| Time | Variables | Total group  n = 21 | G1  n = 9 | G2  n = 12 |
| --- | --- | --- | --- | --- |
| T0  baseline | LBM, kg | 21.70 (16.30 – 28.20) | 21.70 (16.30 – 28.70) | 21.40 (17.70 – 25.80) |
|  | FM, kg | 8.06 (3.52 – 26.50) | 5.91 (2.00 – 8.06) | 20.10 (5.00 – 30.50) |
| T1  pre-intervention |  |  |  |  |
|  | LBM, kg | 21.30 (17.50 – 25.80) | 21.80 (17.70 – 27.30) | 20.80 (17.30 – 24.40) |
|  | FM, kg | 8.12 (4.52 – 25.40) | 5.47 (2.07 – 8.72) | 16.20 (7.19 – 31.10) |
|  |  |  |  |  |
| T2  post-intervention | LBM, kg | 22.10 (17.70 – 26.20) | 22.00 (17.70 – 26.90) | 23.30 (17.80 – 25.90) |
|  | FM, kg | 10.10 (5.02 – 27.40) | 5.02 (1.60 – 8.85) | 22.40 (9.71 – 32.60) |

LBM: lean body mass; FM: fat mass; G1: patients with zinc deficiency; G2: patients with adequate zinc. Results are shown as median and interquartile ranges (25-75%).
